# Microfluidic Assembly of Poly(glutamic acid) Nanogels Through SPAAC Click Chemistry

**DOI:** 10.3390/pharmaceutics17091150

**Published:** 2025-09-02

**Authors:** Pasquale Mastella, Stefano Luin

**Affiliations:** 1 NEST Laboratory, Scuola Normale Superiore, Piazza San Silvestro 12, 56127 Pisa, PI, Italy; 2 Fondazione Pisana per la Scienza ONLUS, Via Ferruccio Giovannini 13, 56017 San Giuliano Terme, PI, Italy; 3 NEST Laboratory, Istituto Nanoscienze-CNR, Piazza San Silvestro 12, 56127 Pisa, PI, Italy

**Keywords:** nanogel, Poly(γ-glutamic acid), click chemistry, strain-promoted azide–alkyne cycloaddition (SPAAC), drug delivery, doxorubicin encapsulation

## Abstract

**Background/Objectives:** Nanogels (NGs) are promising carriers for drug delivery due to their tunable size, biocompatibility, and capability to encapsulate sensitive molecules. However, conventional batch synthesis often lacks control over key parameters, such as size distribution and encapsulation efficiency. This study aimed to develop a microfluidic platform for the reproducible synthesis of poly(α-glutamic acid) (PGA)-based NGs using strain-promoted azide–alkyne cycloaddition (SPAAC) click chemistry and to investigate the effects of flow parameters on the physicochemical properties of nanogels. **Methods:** Functionalized PGAs (with azide and DBCO) were co-injected into a microfluidic system within a flux of acetone to form NGs via SPAAC. Flow rate ratios (FRR) and total flow rates were systematically screened at 25 °C, with tests at 50 °C. We evaluated the particle size, polydispersity index (PDI), zeta potential, and encapsulation efficiency (EE%) of doxorubicin-loaded NGs. **Results:** NGs with tunable sizes ranging from ~50 nm to >170 nm and low PDI (<0.1 in optimal conditions) were obtained. Higher FRR and total flow rates yielded smaller and more uniform NGs. Doxorubicin loading did not affect the nanogel size and uniformity, and in some cases, it improved them. The EE% reached up to ~65%, and ~40% for the best formulations. Elevated temperature improved the characteristics of drug-loaded nanogels at intermediate solvent ratios. Compared to batch synthesis, the microfluidic process offers enhanced reproducibility and size control. **Conclusions:** Microfluidic SPAAC synthesis enables precise and scalable fabrication of PGA NGs with controllable size and drug loading. This platform supports future integration of on-chip purification and monitoring for clinical nanomedicine applications.

## 1. Introduction

In recent years, numerous strategies have been developed for the synthesis of nanogels (NGs), reflecting their broad applicability in drug delivery, diagnostics, and biotechnology [1]. These three-dimensional nanoscale hydrophilic polymeric networks offer unique advantages, such as high water absorption capacity, biocompatibility, and functional versatility. Both natural polymers, including polysaccharides and proteins, and synthetic polymers have been employed to produce NGs with tailored characteristics, such as particle size, colloidal stability, and the ability to encapsulate a wide range of therapeutic molecules (e.g., small drugs, proteins, and nucleic acids) and release them in a controlled manner in response to specific physiological stimuli, including changes in pH, temperature, ionic strength, or enzymatic activity. The most commonly used polymers are chitosan, hyaluronic acid, and dextran or derivatives, each possessing distinct physicochemical and biological properties that favor their use in various clinical contexts [2,3,4]. The development of nanostructured drug delivery systems requires precise control over key parameters, such as size, internal structure, and chemical composition. This control is essential for optimizing therapeutic efficacy, biodistribution, and safety. Although conventional batch synthesis is widely used, it often suffers from poor control over particle size distribution, low encapsulation efficiency, and batch-to-batch variability. These limitations are especially critical when designing nanocarriers for biomedical applications, where uniformity and reproducibility are essential [5]. Therefore, the development of alternative strategies that allow precise control over the synthesis parameters has become a major area of interest.

Flow chemistry, also known as continuous processing, involves the continuous pumping of reagents through microreactors, enabling reactions to occur under steady-state conditions [6]. Compared with batch synthesis, this approach offers superior control over the reaction parameters, improved safety, enhanced mass and heat transfer, and increased scalability. These characteristics make flow chemistry particularly well-suited for the synthesis of soft nanomaterials like NGs [7], which benefit from mild and controllable reaction environments. Importantly, flow reactors provide the opportunity to exploit synthesis routes that are difficult to reproduce in batch, such as those requiring rapid temperature cycling or hazardous intermediates, in a safe and reproducible manner. Small reactor volumes, excellent heat exchange surfaces, and efficient mixing allow for better selectivity, improved thermal control (e.g., for isothermal operation), and real-time process control [8]. In addition, the modular nature of flow chemistry systems allows for the easy integration of multiple synthesis steps in a continuous sequence, further increasing the efficiency and scalability of the process [9].

Microfluidic technology represents a specialized subfield of flow chemistry, enabling the manipulation of fluids in channels with dimensions typically ranging from tens to hundreds of micrometers [10]. This miniaturization allows for reduced reagent consumption, lower waste production, and better compatibility with automation and high-throughput screening. Microfluidics has demonstrated great potential in the synthesis of organic and inorganic nanoparticles [11,12], including metal-organic frameworks [13,14], and, in particular, of organic NGs [15], owing to its ability to generate highly uniform nanostructures with narrow size distributions, high encapsulation efficiencies, and more controlled release profiles [16]. This is due to the confined geometries, possibility of laminar flow conditions, and continuous and highly reproducible nature of microfluidic manufacturing, which enables precise control over mixing regimes, leading to improved reproducibility and scale-down feasibility for early-stage formulation screening [11]. Several microfluidic configurations have been developed for nanoparticle and nanogel production, including hydrodynamic flow focusing (HFF) [17] and staggered herringbone micromixers (SHM) [18]. HFF leverages the laminar nature of microfluidic flows to create a narrow central stream of a nanoparticle precursor, flanked by streams of an antisolvent (a fluid in which the precursor is poorly soluble), thus promoting controlled precipitation and self-assembly. In this setup, mixing occurs via diffusion at the interface between the streams, allowing for control over nucleation and particle growth. In contrast, SHM introduces microstructures within the channels, which induce chaotic advection and enhance mixing efficiency. This passive micromixing technique reduces synthesis time and minimizes dilution effects. Furthermore, active micromixing techniques, such as those based on ultrasonic waves [19], have been employed to accelerate mixing, enhance nucleation rates, and reduce particle size and polydispersity. The ability to combine passive and active mixing strategies offers a high level of versatility for tuning the characteristics of NGs.

These approaches provide not only greater reproducibility but also adaptability for encapsulating sensitive biological cargoes under mild conditions; for these reasons, microfluidics has emerged as a powerful tool in drug delivery. For example, several studies have shown that microfluidic synthesis can yield NGs with narrower size distributions and more consistent internal architectures, enabling tunability in drug release profiles, compared to conventional batch methods [11,12,20,21]. As already stated, microfluidic systems also facilitate real-time monitoring and data acquisition, which are critical for establishing robust quality control protocols in pharmaceutical production [22]. Despite these advantages, challenges such as channel clogging, limited solvent compatibility, and material absorption (e.g., with PDMS devices) must be addressed to ensure long-term reliability and scalability [23,24]. To further enhance the structural precision, reproducibility, and functional versatility of NGs synthesized via microfluidics, the incorporation of advanced, highly selective crosslinking chemistries beyond conventional approaches is highly advantageous. Indeed, the combination of microfluidic technology with click chemistry strategies has been explored for microgel production [25,26]. These studies demonstrated the reproducible synthesis of microgels with tunable mechanical and structural properties under mild conditions, which is particularly advantageous for encapsulating sensitive biological payloads, such as proteins or nucleic acids. Nonetheless, this approach remains underexplored for NGs production.

Click chemistry encompasses a class of highly selective modular reactions that proceed under mild conditions and yield stable products [27]. Among the various crosslinking strategies, strain-promoted azide–alkyne cycloaddition (SPAAC) is notable for its efficiency, bioorthogonality, and catalyst-free reaction conditions [28]. Thus, the integration of SPAAC within microfluidic platforms represents a strategic approach for the reproducible and efficient production of functional nanomaterials. This synergy enables precise spatial and temporal control over crosslinking, contributing to improved homogeneity, enhanced stability, and tunable degradation rates of the resulting NGs.

In this work, we report a microfluidic approach for the synthesis of NGs based on poly(α-glutamic acid) (PGA) crosslinked via SPAAC chemistry. PGA is a water-soluble, biodegradable, and biocompatible polymer known for its ability to encapsulate hydrophilic drugs [29]. Its tunable chemical structure and favorable biological profile make it an ideal candidate for developing targeted drug delivery systems [30]. Here, we investigate mostly the effect of flow rate ratios within the microfluidic device on the physicochemical properties of the resulting NGs. We demonstrate that by modulating only these fluidic conditions, it is possible to reproducibly tune the size and morphology of NGs, offering a promising route for formulation optimization and process scalability. Furthermore, our platform lays the groundwork for the future integration of additional on-chip functionalities, such as purification, real-time characterization, or multi-step synthesis workflows, aimed at producing next-generation therapeutic NGs.

## 2. Materials and Methods

### 2.1. Materials

All reagents used in this study were of analytical or reagent grade and were used as received unless specified otherwise. Poly(L-glutamic acid) sodium salt (PGA, MW 15,000–50,000) was obtained from Sigma-Aldrich(St. Louis, MO, USA). Azide-functionalized PGA (PGA-N_3_, MW 34,000, 10% azide-modified side chains) was purchased from Alamanda Polymers, Inc. (Huntsville, AL, USA). Dibenzocyclooctyne-amine (DBCO-NH_2_) and doxorubicin hydrochloride (Dox·HCl) were acquired from MedChemExpress (South Brunswick, NJ, USA). The carbodiimide activator N-(3-dimethylaminopropyl)-N′-ethylcarbodiimide (EDC) was supplied by Sigma-Aldrich. All solvents used in this study were of analytical quality. Ultrapure water (Milli-Q system; Merck Millipore, Darmstadt, Germany) was used to prepare all aqueous solutions. NGs purification procedures involved the use of MicroSpin G-50 Columns (GE Healthcare, Chicago, IL, USA) and 0.45 μm nylon centrifugal filters (VWR^®^, Radnor, PA, USA), operated according to the respective manufacturers’ protocols.

### 2.2. Synthesis of DBCO-Functionalized PGA

As previously described in detail and characterized in our previous work [31], the functionalization of PGA with dibenzocyclooctyne groups (DBCO) was carried out using carbodiimide chemistry. Briefly, 100 µL of poly(L-glutamic acid) (MW 15–50 kDa) was dissolved at a concentration of 10 mg/mL in MES-buffered saline (MBS, pH 6.5). To activate the carboxyl groups, EDC was added in a 1.5-fold molar excess with respect to the glutamate residues, and the reaction mixture was incubated at room temperature for 20 min with gentle stirring. DBCO-NH_2_ was prepared as a 70 mg/mL solution in DMSO and added to the activated polymer solution. Different feed ratios of DBCO-NH_2_ were tested to modulate the extent of functionalization: a 14 mol% feed ratio relative to glutamate monomers resulted in ~10% substitution, whereas a 30 mol% input yielded ~20% functionalization. The coupling reaction was allowed to proceed for 4 h at room temperature with continuous agitation. To eliminate unreacted DBCO-NH_2_, the reaction mixture was purified using 10 kDa molecular weight cut-off Amicon^®^ Ultra centrifugal filters. The sample was centrifuged at 8000× *g* for 10 min at room temperature and washed 3 to 5 times with ultrapure water. The successful attachment of DBCO moieties to the PGA backbone was confirmed by high-performance liquid chromatography (HPLC) using a Dionex Ultra 3000 system equipped with a PolySep-GFC-P 4000 analytical column and a corresponding guard column. The mobile phase consisted of phosphate-buffered saline (1×, pH 7.4). The incorporation of DBCO was quantified by measuring the absorbance at 310 nm using UV-Vis spectroscopy.

### 2.3. Configuration of the Microfluidic System Setup

**Figure 1:**
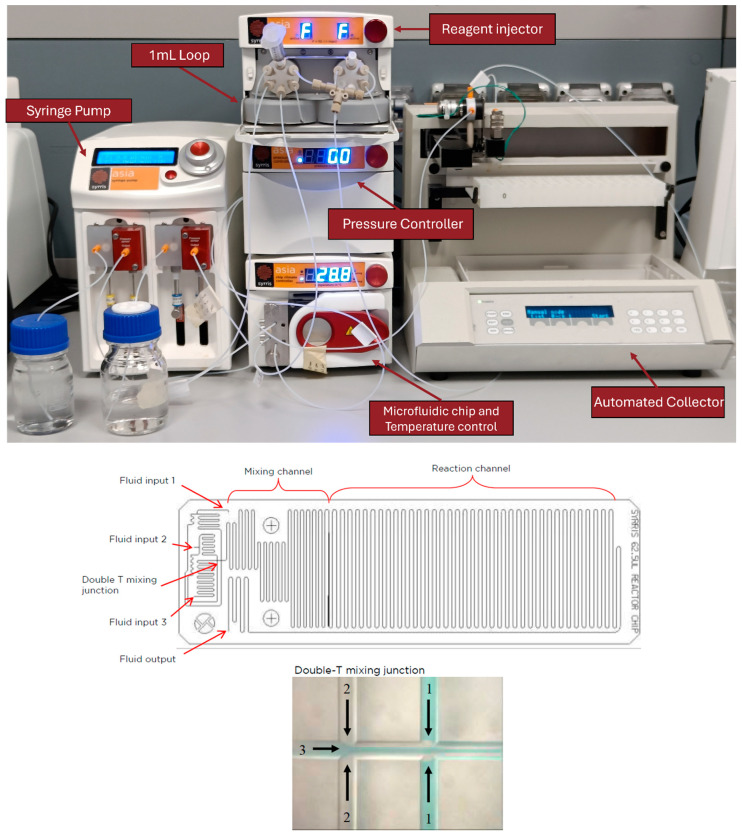
The image shows (**top**) the Asia Syrris system equipped with all modules used for NGs synthesis, including syringe pumps, reactor injector, temperature controller, heating module, and fraction collector. At the (**bottom**), a schematic of the microfluidic chip is shown, highlighting the fluidic inputs, mixing channel, reaction channel, and double-T mixing junction where the three input streams converge. (Image source: Syrris, from “Asia Tech Sup Note—Microreactors datasheet CUSTOMER RELEASE”, p. 4. © Syrris Ltd.).

NGs synthesis was carried out using a modular automated flow chemistry platform (Asia, Syrris Ltd., Royston, UK) designed to ensure controlled fluid mixing, temperature regulation, and automated sample collection. The system is composed of a syringe pump module with two independent channels (Pump A and Pump B), a reagent injector equipped with a six-port valve and a 1 mL loop, a glass microfluidic chip (250 μL internal volume, three inlets) inserted into a temperature-controlled Chip Climate Controller, a backpressure regulator, and an automated collector (Figure 1). Pump A was loaded with ultrapure water and connected to the reagent injector, which was connected to a 1 mL loop. This was preloaded with a solution containing the polymers of interest (see next section), which was then transferred to inlet 3 of the microfluidic chip. Pump B, containing acetone, was connected directly to inlet 1 of the chip. Inlet 2 was sealed with a cap because it was not used during the experiments. The outlet of the chip was connected in series to the backpressure controller and then to the automated collector. The chip was operated at controlled temperatures of 25 °C or 50 °C, depending on the experimental conditions (Table 1). All tubing and wettable components are made of polytetrafluoroethylene (PTFE), selected for its high chemical resistance and compatibility with both aqueous and organic phases. The fluidic path was assembled using PTFE tubes with defined lengths and internal diameters (ID) to regulate residence time and flow profiles. Specifically, the segment from Pump A to the injector is 611 mm long with an ID of 0.5 mm; from Pump B to the chip, 614 mm with an ID of 0.3 mm; from the injector to the chip, 509 mm with an ID of 0.5 mm; from the chip outlet to the backpressure controller, 611 mm with an ID of 0.5 mm; and from the backpressure controller to the collector, 710 mm with an ID of 0.3 mm.

Through this configuration, precise and reproducible mixing under laminar flow conditions is achieved, allowing for accurate and scalable nanoparticle formulation.

### 2.4. Microfluidics-Assisted Inverse-Nanoprecipitation

NGs were formulated using the microfluidic system described in the previous section. The two polymers, azide-functionalized polyglutamic acid (PGA-N_3_) and DBCO-modified PGA (PGA-DBCO), were premixed and diluted in ultrapure water to a final concentration of 0.5% *w*/*v*. This polymer mixture was loaded into a syringe and injected through one of the ports of a six-port valve to fill a 1 mL loop of the reagent injector. Meanwhile, Pump A and Pump B of the syringe pump module of the system were loaded with Milli-Q water and acetone, respectively. Flow rates were varied systematically to screen different conditions, as reported in Table 1. The resulting NG dispersions were collected into Eppendorf tubes using an automated fraction collector connected to the chip outlet. Samples were formulated at two different temperatures (25 °C and 50 °C), as regulated by the chip climate controller. Although the synthesis procedure remained identical, the post-synthesis treatment varied depending on the temperature condition. The samples synthesized at 25 °C were sealed and agitated in a thermomixer at 25 °C and 500 rpm for at least 5 h to ensure complete crosslinking. Subsequently, the tubes were placed under a chemical fume hood and allowed to evaporate overnight with continuous agitation under the same conditions. In contrast, samples synthesized at 50 °C were immediately agitated in a thermomixer at 50 °C and 500 rpm under a fume hood for 2 h to allow solvent evaporation. After synthesis and post-treatment, NGs were filtered through 0.45 μm nylon membrane filters and resuspended in PBS 1×. Purified samples were stored at 4 °C until further characterization. The same synthesis protocol was used to formulate doxorubicin (Dox)-loaded nanogels (NGs Dox). In this case, Dox was added to the polymer mixture during the premixing step before loading into the loop at a drug/polymer mass ratio of 1:50. The flow conditions and post-synthesis processing were identical to those described previously. However, the purification procedure for the drug-loaded NGs included an additional size-exclusion step using MicroSpin G-50 columns to remove unencapsulated Dox. The purified samples were then stored at 4 °C until further characterization.

### 2.5. Experimental Design

The experimental design involved systematic variation of the flow conditions within the microfluidic system to optimize NGs formation and investigate their effects on the physicochemical properties of the particles. Specifically, the solvent streams of acetone and Milli-Q water had varying flow rates, resulting in different flow rate ratios (FRR) and total flow rates (flux) inside the chip mixing and reaction channels, as listed in Table 1. The flow rate ratio, defined as the volumetric flow rate of acetone to that of water, varied from 1 to 7 to explore a wide range of solvent environments and their effects on polymer self-assembly and crosslinking kinetics. For each FRR, total flow rates ranging from 100 to 300 mL/min were tested to assess the impact of shear forces and residence time on the size and uniformity of the NGs. To ensure stable and reproducible flow conditions, each experiment was initiated with the injection of a priming volume (50 µL), introduced prior to the polymer solution to stabilize flow within the microfluidic channels and allow the system to reach steady-state conditions. At the end of each run, a flushing segment of equal volume (50 µL) was injected to clear the channels and minimize the transient effects of residual reagents. These volumes were composed of the same solvents used in the main experiment and were injected through the same inlet channels as the reaction fluid. The resulting NG suspensions were collected automatically using an integrated fraction collector connected to the chip outlet. This setup allowed for continuous, hands-free sampling, reducing the risk of contamination and ensuring consistent sample volumes across experiments. The collected samples were then subjected to downstream processing, including agitation for complete crosslinking, acetone evaporation, and purification. This comprehensive screening approach allowed the identification of optimal flow parameters that balance efficient mixing, reaction kinetics, and particle stability. The data generated guided subsequent optimization steps and provided insights into the relationship between microfluidic flow conditions and NGs characteristics, such as size distribution and encapsulation efficiency.

### 2.6. Physicochemical Characterization

#### 2.6.1. Particle Size and Polydispersity

The hydrodynamic diameter and polydispersity index (PDI) of the NGs were assessed via Dynamic Light Scattering (DLS) using a Zetasizer Nano ZS (Malvern Instruments Ltd., Malvern, UK) equipped with a 633 nm laser and operating at a backscattering angle of 173°. NG samples were diluted in 1× PBS to a final concentration of 5 mg/mL, briefly sonicated (60 s), and filtered using 0.45 µm nylon membrane filters (VWR^®^) prior to analysis. Three independent batches of each formulation were prepared and measured. Each batch was analyzed in triplicate with five consecutive acquisitions per run. Reported values for mean diameter (Z-Average) and PDI were calculated using the cumulant method and are expressed as mean ± standard deviation (SD). Instrument settings for laser attenuation and beam alignment were automatically optimized before each measurement.

#### 2.6.2. Zeta Potential

The zeta potential (ζ) of NGs was measured using the same Zetasizer Nano ZS instrument (Malvern Instruments, UK) operating at 25 °C with a 633 nm laser and disposable folded capillary cells. Samples were diluted in 0.1× PBS (pH 7.4) and filtered through 0.45 μm nylon membrane filters (VWR^®^) before analysis. Three independent replicates were analyzed for each formulation, with five measurements taken per replicate. Electrophoretic mobility was determined and automatically converted to zeta potential values using the instrument software (Malvern Zetasizer, Version 7.13, Malvern Instruments Ltd. Malvern, UK). Results are expressed as mean ± standard deviation.

#### 2.6.3. Drug Encapsulation

To quantify the Dox HCl encapsulated within the NGs, the drug was added directly to the polymer mixture prior to injection, as previously described. A fixed drug-to-polymer mass ratio of 1:50 was maintained. After formulation via the microfluidic protocol, the resulting dispersion underwent solvent evaporation, followed by purification using MicroSpin G-50 columns to remove unencapsulated drugs and low-molecular-weight species. Purified NGs were resuspended in 1× PBS to a final volume of 100 μL. Dox content was determined via ultraviolet-visible (UV-Vis) spectrophotometry at 480 nm using a Cary 3500 instrument (Agilent Technologies) with 1 cm path-length quartz cuvettes. Quantification was performed using a calibration curve constructed with Dox standards prepared in ultrapure water, which showed linearity in the range of 0.78 to 50 μg/mL (Figure S7 in the ESI of [31]). To determine the nanogel mass, samples were lyophilized after resuspension in PBS 1× containing trehalose (10 mg/mL). The resulting dry product was weighed using an analytical balance (Sartorius Cubis^®^). To calculate the drug loading (DL%), the measured lyophilized mass was corrected by subtracting the known amounts of trehalose and residual PBS salts introduced with the suspension. The encapsulation efficiency (EE%) and drug loading capacity (DL%) were calculated using the following equations:

$$EE\left(\%\right)=\frac{Mass\;of\;Drugs\;encapsulated}{Total\;mass\;of\;Drugs\;added}\times 100$$

$$DL(\%)=\frac{Mass\;of\;Drugs\;encapsulated}{Mass\;of\;Nanogel}\times 100$$

### 2.7. Data Analysis

To analyze the dependence of size, PDI, and EE% on FRR and flux, we employed a non-parametric interpolation approach based on Thin Plate Spline (TPS) fitting implemented in R (v4.5.0) using the “fields” package (v16.3.1); this method smooths and interpolates multidimensional data. After data cleaning, aggregation, and averaging, the model was evaluated over a regular grid to generate three-dimensional response surfaces, enabling a continuous representation of trends across the experimental space. Graphics were produced using R and GraphPad (version 8.0, GraphPad Software, San Diego, CA, USA).

## 3. Results

### 3.1. Optimization Strategy for Microfluidic NGs

The optimization of NGs synthesis in the microfluidic system involved a systematic exploration of key process parameters to understand their effects on nanoparticle size and polydispersity. A full factorial matrix comprising four total flow rates and six acetone-to-water volume ratios (*n* = 24) was evaluated. This matrix was described as quasi-orthogonal, meaning it was designed to approximate an ideal orthogonal experimental design in which factors varied independently and without correlation. Although some minor correlations or variations were present due to practical constraints, such as slight fluctuations in flow rates, the arrangement still allowed reliable estimation of the effects of individual factors and their interactions. The selection of solvent ratios was initially guided by the conditions previously used in the batch formulation, where specific acetone-to-water ratios (5:1) yielded stable NGs [31]. Based on these reference conditions, a wider range of solvent ratios was tested to evaluate the influence of microfluidic mixing on particle formation. A similar rationale was adopted for the total flow rate, which varied from 100 to 300 µL/min to represent different mixing intensities. The solvent ratio plays a crucial role in determining the local concentration gradients and the mixing conditions during nanoprecipitation, which in turn affect nucleation and particle growth. By adjusting this parameter, it is possible to modulate the nanoparticle size and uniformity, which are key features for high-quality NGs. Although the total flow rate was not strictly constant across all solvent ratios, the data matrix remained sufficiently structured to allow for a two-factor response surface analysis [32]. The total flow rate was treated as a continuous variable, and no irregular trends due to minor flow variations were observed. To analyze the experimental data, we used the TPS method, which performs simultaneous smoothing and interpolation, allowing the modeling of complex, potentially nonlinear relationships between microfluidic process parameters, such as total flow rate and solvent ratio, and the resulting NGs properties, without requiring a predefined functional form. This approach supports an intuitive understanding of system behavior and facilitates the prediction of outcomes under untested conditions, providing an efficient, data-driven pathway for process optimization without extensive experimentation.

### 3.2. Influence of Flow Parameters on NGs Characteristics

#### 3.2.1. Effect on Particle Size

Regulating the size of NGs plays a fundamental role in the development of drug delivery systems, as the particle dimensions directly impact transport through biological barriers, internalization by cells, residence time in circulation, and kinetics of drug release. In this study, NGs were specifically designed as potential drug carriers, and their sizes were optimized accordingly. Although no strict target size was imposed, particles below 200 nm were considered preferable to ensure efficient behavior in vivo. Among other benefits, smaller and stable NGs are known to accumulate more effectively in tumor tissues through the Enhanced Permeability and Retention (EPR) effect, making sub-200-nm sizes, and in particular sizes around 100 nm or slightly lower, attractive for systemic administration.

**Figure 2:**
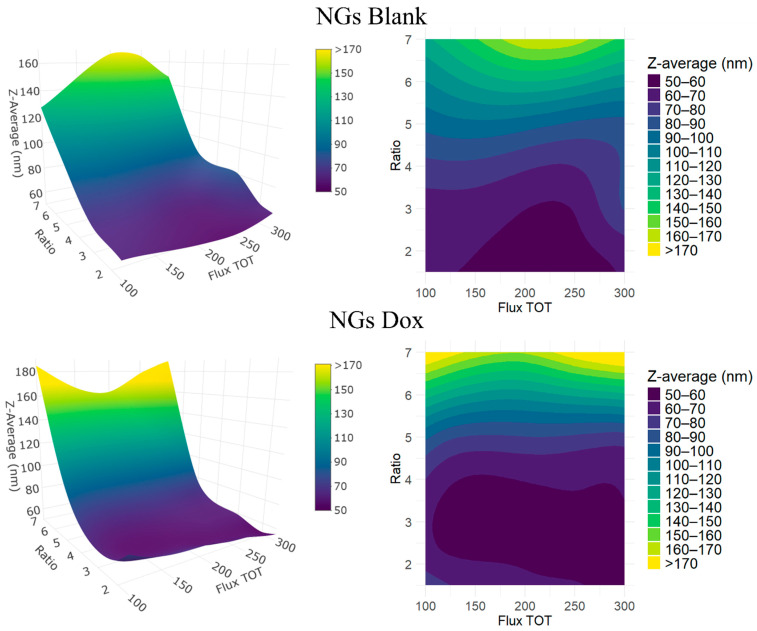
Response surfaces (**left**) and contour plots (**right**) showing the experimental effects of the acetone-to-water ratio (Ratio) and total flow rate in mL/min (Flux TOT) on the Z-average diameter (size, color-coded) of NGs. Top: NGs Blank; Bottom: NGs Dox.

NGs sizes were evaluated as a function of the acetone-to-water volume ratio (ranging from 1:1 to 7:1) and the total flow rate (100–300 µL/min), as summarized in Table 1 and Table 2 and depicted in the response surface plot (Figure 2). The contour map clearly reveals how particle size is jointly influenced by both parameters, outlining a region of optimal conditions and highlighting critical transitions, where small changes in solvent ratio or flow rate lead to abrupt variations in particle size. The highest acetone-to-water ratio tested (7:1) generated significantly larger NGs across all flow rates, with average sizes reaching or exceeding 140 nm. This trend, also reflected in the yellow-green band dominating the top region of the plot, suggests that an excessive amount of organic solvent perturbs the desolvation process, leading to inefficient nucleation and/or less-compact polymer networks. Under these conditions, the local composition, that is, the instantaneous acetone-to-water ratio at the precise site of polymer precipitation, may not support controlled nucleation and network formation. This transient solvent environment can negatively affect both the kinetics of polymer precipitation and the subsequent click-crosslinking reaction, resulting in the formation of bulkier and more heterogeneous particles. These formulations appear to fall outside the optimal processing window for controlled NGs formation via SPAAC under the tested microfluidic conditions. In contrast, intermediate solvent ratios from 5:1 to 2:1 exhibited more favorable behavior. These ratios supported the formation of smaller NGs, particularly when combined with higher total flow rates. The central band of the contour plot shows a progressive size reduction from approximately 100 nm to around 60 nm as the total flow rate increases from 100 to 300 µL/min, confirming the effect of the formulation on the hydrodynamic radius. Among these, the 3:1 and 2:1 ratios produced the smallest particles overall (as low as ~56 nm), indicating an ideal balance of organic and aqueous phases that promoted fast and homogeneous precipitation. Interestingly, the 1.5:1 ratio, despite maintaining a relatively low size range, showed more variability and a tendency toward slightly larger particles than 2:1. This suggests a possible lower limit for the organic content required for effective desolvation. Finally, the 1:1 ratio systematically failed to generate stable NGs, with all samples exhibiting visible precipitation or aggregation. These conditions likely fall below the critical organic content threshold necessary to induce polymer collapse and network formation.

An increase in the total flow rate generally resulted in smaller particles across nearly all viable ratios. This observation is attributed to enhanced mixing efficiency within the microfluidic device, which reduces mixing time and promotes uniform supersaturation. Faster flows favor instantaneous nucleation over diffusion-limited growth, thereby reducing the final particle size. This trend is especially pronounced for intermediate solvent ratios, where the interplay between flow rate and solvent polarity is finely balanced. The effect is not reproduced at the extremes (1.5:1 and 7:1), where either insufficient desolvation or excessive organic content predominates, reducing the influence of flow rate on NGs formation.

NGs were also synthesized in the presence of Dox to investigate the influence of drug encapsulation on particle size. Interestingly, NG Dox formulations at intermediate FRR tended to have slightly smaller hydrodynamic diameters than their blank counterparts, with an average size reduction of ~5%. However, this behavior was not entirely consistent across all conditions; even at the same acetone-to-water ratio, NGs prepared at different total flow rates occasionally showed divergent trends, with drug-loaded particles being larger than their blank counterparts in some cases. For instance, at a 3:1 acetone-to-water ratio and 250 µL/min total flow rate, NGs Blank measured ~56.5 nm, while their NGs Dox analogues showed a slightly larger size, about 61.8 nm. This confirms that the flow rate, and thus the overall process dynamics, plays a critical role beyond the simple compositional effects. The observed trend suggests that doxorubicin also plays an active role in NGs formation, likely promoting a more compact polymer conformation. A possible explanation for this lies in the electrostatic interactions between the cationic amine groups of Dox and the anionic carboxylate residues of the polyglutamic acid backbone. These interactions may act as nucleation centers, fostering rapid and localized polymer collapse, thereby reducing the size and increasing the density of the network. In this scenario, the drug molecule behaves not merely as a passive payload but as an integral component of the self-assembly process, enhancing NGs compaction and possibly influencing crosslinking topology. This phenomenon appears to be formulation-dependent but robust, as size reduction was observed across various solvent ratios and flow rates. Importantly, the presence of Dox did not compromise colloidal stability or significantly shift the optimal formulation window. Sub-100 nm sizes were consistently achieved in the 3:1–2:1 ratio range, confirming the versatility and efficiency of the system, even under drug-loading conditions.

Finally, the residence time within the microfluidic channel, which depends on the internal volume of the chip (250 µL) and the total flow rate, contributes to the observed size trends. Shorter residence times at higher flow rates (e.g., ~50 s at 300 µL/min) favor rapid mixing and nucleation, limiting the window for particle growth and leading to smaller and more uniform NGs. In contrast, longer residence times (e.g., ~150 s at 100 µL/min) may promote further growth and broadening of the size distribution, especially under non-optimal solvent conditions. Thus, the observed synergy between solvent composition and flow rate may be partially mediated by the kinetics of self-assembly under confined flow conditions.

#### 3.2.2. Effect on Polydispersity Index

While particle size screening provides important insights into how formulation parameters influence NGs dimensions, the polydispersity index (PDI) reflects a different aspect of the system: the uniformity of the particle population, which is crucial for reproducibility and predictable biological behavior. In microfluidic synthesis, low PDI values are generally expected due to the enhanced and highly controlled mixing conditions that promote uniform nucleation and growth. Table 2 also lists the PDI values for NGs Blank and NGs Dox across the explored acetone-to-water ratios and flow rates. The NGs generally showed low PDI values, indicating a narrow size distribution and good formulation quality under most of the tested conditions. Dox loading, for this parameter, seems not to have a striking effect, with NGs Dox samples exhibiting comparable PDI values to blank ones, except maybe at the extreme FRR conditions (1.5:1 and 7:1), where Dox seems to improve the reproducibility of NGs formation.

**Figure 3:**
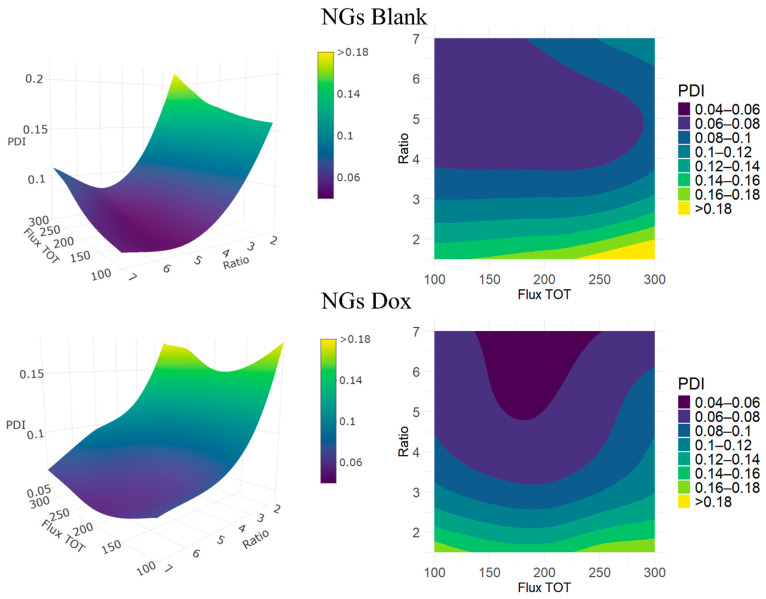
Response surfaces (**left**) and contour plots (**right**) showing the experimental effects of acetone-to-water ratio (Ratio) and total flow rate in mL/min (Flux TOT) on the PDI (Z-axis, color-coded) of nanogels (NGs). Top: NGs Blank; Bottom: NGs Dox.

Figure 3 shows the influence of the process parameters on the PDI. For both NGs Blank and NGs Dox, the lowest PDI values are observed at low to intermediate flow rates (100–200 µL/min) and acetone-to-water ratios around 5:1–7:1. These conditions likely offer an optimal balance between mixing and desolvation, promoting uniform nucleation and efficient crosslinking. In the NGs blank formulations (Figure 3, top), the lowest PDI values (~0.05) were consistently observed at an FRR of 5:1 across multiple flow rates. Outside this region, especially at low solvent ratios (e.g., 1.5:1) and high flow rates, the PDI increased markedly (up to 0.32), suggesting less controlled formation and broader size distributions. These extreme conditions were avoided in further tests due to issues like poor mixing or the risk of clogging from precipitates.

NGs Dox (Figure 3, bottom) showed lower PDI values under specific conditions (as low as 0.05 at FRR 7:1 and 200 µL/min). However, the formulation showed more variability overall, with PDI values of up to 0.14 under other conditions at the same ratio. Although the interpolated contour plot may visually suggest a broader low-PDI region for the Dox formulation, the experimental data indicate that Blank NGs and Dox NGs achieved low PDI values (<0.10) across a similar number of conditions, with the former having lower PDIs, particularly for the 5:1 FRR. This suggests that the presence of the drug may not universally improve the formulation uniformity and may slightly increase the polydispersity under certain flow regimes. The lowest PDI was obtained at an FRR of 7:1 and a flux of 200 µL/min. The bottom graphs in Figure 3 seem to indicate that the PDI would decrease even more at higher FRR; however, we have shown in the previous section that at high FRR, the NGs become too big.

In summary, the NGs exhibited excellent size homogeneity, except at the lowest FRR, with consistently low PDI values, confirming the robustness and reproducibility of the microfluidic click-chemistry formulation method. This uniformity reflects the efficiency of polymer chain desolvation and crosslinking under optimal conditions, resulting in narrowly dispersed particle populations.

#### 3.2.3. Effect on Encapsulation Efficiency

**Figure 4:**
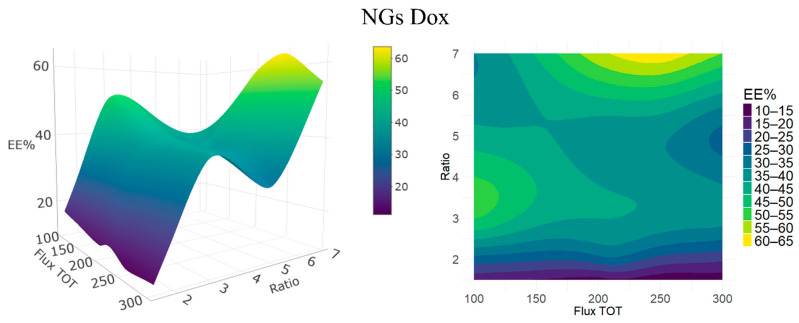
Response surface (**left**) and contour plot (**right**) showing the experimental effects of the acetone-to-water ratio (Ratio) and total flow rate in mL/min (Flux TOT) on the encapsulation efficiency (EE%, color-coded) of NGs Dox.

In drug delivery applications, achieving a high encapsulation efficiency (EE%) is important for improving process efficiency, reducing formulation costs, and minimizing waste, which are key factors in the development of scalable and sustainable nanocarriers. The encapsulation efficiency (EE%) represents the proportion of drug successfully entrapped within the NGs matrix relative to the total amount initially used. We did not measure the DL% (the weight ratio of drug loaded in the nanoparticles over their weight) for each formulation; however, whenever we performed such measurements, we measured a quite constant low loss of polymers around 20%, most probably due to the purification steps. This, together with the fact that we used the same initial quantities of materials for each formulation, means that the observed differences in EE% also reflect variations in DL%. In Figure 4, we show contour and surface plots similar to those in Figure 2 and Figure 3 to illustrate trends in EE%. The behavior in this case is more complicated, with two local maxima: at the highest FRR and intermediate total flux (~200–250 µL/min) and for low fluxes at intermediate ratios. In the following discussion, we focus mostly on the results summarized in Table 2.

The highest EE% values, reaching up to approximately 60–65% were obtained at the highest tested solvent ratio (7:1) and at high flow rates (around 250 µL/min). Under these conditions, drug entrapment is likely enhanced due to the formation of significantly larger NGs (average size >170 nm), as discussed in the previous section on particle size. Larger NGs tend to provide not only greater internal volume and a higher volume-to-surface ratio, but also a denser polymeric network, resulting in a more compact and tightly crosslinked structure, which can enhance drug retention. It should be noted that the relationship between particle size and network density is strongly influenced by the specific synthesis conditions and polymer chemistry. These factors influence the degree of crosslinking and porosity, which ultimately determine the encapsulation efficiency.

Intermediate solvent ratios (5:1 to 3:1) yielded moderate EE% values in the range of 30–50%, with a tendency to increase at lower total fluxes, while maintaining desirable NGs sizes (<100 nm) and low polydispersity. These conditions appear to offer a more balanced trade-off between size, uniformity, and drug loading, making them promising candidates for further studies. Lower solvent ratios (2:1 and 1.5:1) resulted in EE% below 30%, accompanied by smaller particle sizes and an increased PDI. This likely reflects suboptimal desolvation of the polymer phase, which impairs the efficiency of crosslinking via SPAAC chemistry. Limited crosslinking may lead to a polymeric network with increased porosity and weaker structural integrity, thereby reducing the ability of NGs to effectively retain drug molecules. We can notice from this discussion and from Figure 4 that the influence of total flow rate on EE% is solvent-ratio dependent. In addition, the NGs size did not exhibit a simple linear dependence on the flow rate but varied with the solvent composition, emphasizing the importance of jointly optimizing the solvent ratio and flow rate to balance the NGs size and drug encapsulation efficiency. Taken together, these results demonstrate that encapsulation efficiency is highly sensitive to formulation parameters, with solvent composition and flow rate modulating NGs’ size and internal structure, and thus directly influencing the amount of drug retained within the polymeric matrix.

### 3.3. Advanced Optimization: Exploring the Role of Temperature

After completing the full screening of formulation conditions at room temperature, we identified general trends linking solvent ratio and flow rate to NGs size, PDI, and drug loading. While the 4:1 acetone-to-water ratio was not part of the initial matrix, the observed behavior of neighboring conditions (such as 3:1 and 5:1) suggested that 4:1 could represent a promising FRR. Based on this hypothesis, we selected 4:1 as a new formulation condition for testing at elevated temperatures. Although the strain-promoted azide–alkyne cycloaddition (SPAAC) used for NGs formation is a click reaction that proceeds efficiently at room temperature, it remains a chemical process that may benefit from thermal acceleration. An increase in temperature is expected to enhance the reaction kinetics, promote faster network formation, and potentially improve drug retention within the matrix. To investigate this, we conducted a new set of experiments at 50 °C using a 4:1 solvent ratio across a range of total flow rates (100–300 µL/min).

The results showed a clear difference between NGs Blank and NGs Dox produced under the same thermal conditions. NGs Blank exhibited moderately high sizes (~138–180 nm) and acceptable PDI values, but did not show marked improvements compared to the results at room temperature. NGs Dox showed excellent properties, especially at 100 and 200 µL/min, with average sizes around 100 nm, low PDI (0.06–0.1, similar or better than the best formulations at 25 °C), and encapsulation efficiencies reaching up to 40% (and close or a bit higher than the ones obtained for particle of similar sizes and PDI at 25 °C). These findings suggest that elevated temperatures may have a more pronounced impact on the kinetics of NGs formation in the presence of Dox, possibly due to interactions between the drug and the polymer matrix during crosslinking. Although the encapsulation efficiencies at 50 °C were not greater than those observed at room temperature in some formulations, the combination of small size, low PDI, and satisfactory drug loading observed at 100–200 µL/min indicates that thermal modulation can be a useful strategy for fine-tuning NGs properties under specific formulation conditions. This optimization highlights how process parameters, such as temperature, can complement formulation design, offering an additional lever to enhance NGs performance, specifically in drug-loaded systems.

### 3.4. Comparison Between Microfluidic and Batch Synthesis

**Figure 5:**
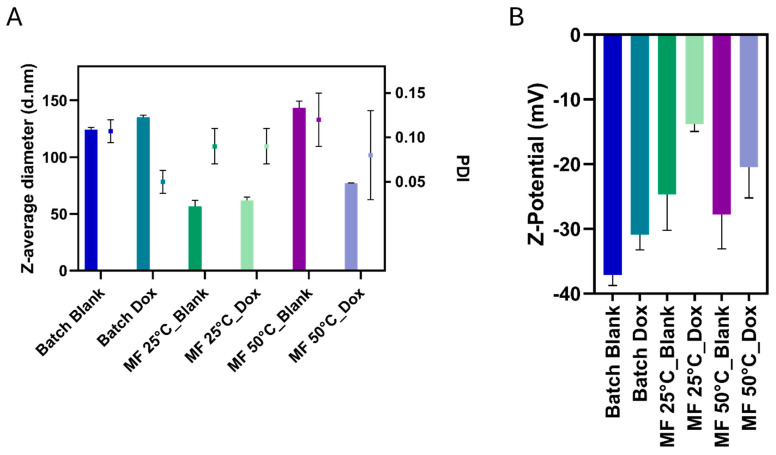
(**A**) Dynamic light scattering analysis of particle size (bars) and polydispersity index (PDI, squares), and (**B**) zeta potential measurements of NGs formulations prepared by batch synthesis (“Batch”), or by microfluidic synthesis (“MF”) at two temperatures, in both blank and DOX-loaded versions. Data represent mean values ± SD from three measurements across three independent batches (*n* = 9).

To explore how the synthesis method affects NGs properties, we compared both blank and NGs Dox formulations previously produced by batch synthesis (Batch) at 25 °C [31] with two representative NGs obtained via microfluidics (MF): condition 10, synthesized at 25 °C, and condition 25, synthesized at 50 °C. These two formulations were selected not only for their favorable size and encapsulation efficiency but also for their low size variability and minimal batch-to-batch differences, which are key indicators of reproducibility in microfluidic synthesis. Although the formulation conditions used for microfluidic synthesis (e.g., solvent ratio) were not identical to those used in the batch process, these microfluidic samples were selected for their optimal size, PDI, and encapsulation efficiency, allowing for a meaningful qualitative comparison. The batch-synthesized NGs, prepared at 25 °C, displayed average sizes of approximately 120 nm (NGs Blank) and 135 nm (NGs Dox), with good control over particle size and a relatively low polydispersity (PDI < 0.15) (Figure 5). Microfluidic synthesis yielded smaller particles with similar PDIs and EE% (around 40% for MF, around 50% for Batch); for instance, the best-performing microfluidic Dox-nanogels produced at 50 °C had an average size of ~77 nm with a PDI of 0.08. Notably, this trend was observed across multiple independent preparations, with minimal variation in size and PDI, confirming the high reproducibility of the microfluidic process and a high level of control over particle formation and size distribution.

In line with the encapsulation efficiencies reported above, the drug loading (DL%) values were 0.90% and 0.83% for microfluidic conditions 10 and 25, respectively, compared to 1.60% for the batch formulation. These relatively low DL% values are consistent with the high polymer-to-drug ratio used in the present experiments, which were designed to investigate the effect of synthesis parameters on particle formation and reproducibility rather than to maximize drug loading. Notably, in our previous work with batch synthesis, a higher drug content was employed (approximately double the amount of doxorubicin), which resulted in correspondingly higher DL% values.

In terms of zeta potential (Figure 5B), batch-synthesized NGs showed values of −37.1 mV for NGs Blank and −30.9 mV for NGs Dox, indicating a mild reduction in charge upon drug loading. Similarly, microfluidic NGs exhibited zeta potentials of −25 mV (blank) and −14 mV (NGs Dox) for syntheses at 25 °C, and −30 mV (NGs Blank) and −25 mV (NGs Dox) at 50 °C. These results suggest that drug loading consistently reduces the absolute value of the charge (i.e., it makes it less negative), likely due to the partial neutralization or shielding of carboxyl groups by the drug. Notably, the microfluidic process at 50 °C better preserved the zeta potential magnitude in the NGs Dox system (−25 mV), possibly as a result of enhanced crosslinking kinetics and denser network architecture, which could increase the ratio of the number of carboxylic groups to the number of Dox molecules while maintaining similar sizes and EE%, thus maintaining a higher absolute zeta potential magnitude. The trend toward lower absolute zeta potentials for MF NGs can also be explained by a similar trend towards smaller sizes, and thus lower ratios of particle volume (approximately proportional to internal negative charge) to hydrodynamic radius, as the zeta potential is directly proportional to the total charge divided by the hydrodynamic radius.

## 4. Discussion

The precise regulation of NGs size is widely recognized in the literature as a fundamental requirement for developing advanced drug delivery systems, as size significantly influences transport across biological barriers, cellular internalization, and drug pharmacokinetics [33]. In our study, the synthesis via microfluidics combined with SPAAC click chemistry allowed for accurate control over the physicochemical properties of NGs by varying the acetone/water ratio and total flow rate. Very high acetone:water ratios like 7:1 tend to generate larger and less homogeneous particles, probably due to inefficient desolvation and less-compact polymer crosslinking, while intermediate ratios (between 5:1 and 2:1), especially 3:1, favored the formation of small and uniform NGs, owing to rapid nucleation and efficient mixing. Increasing the total flow rate was correlated with a decrease in size, attributable to shorter residence times and improved hydrodynamic configuration, which are key elements for synthesis control [32].

Particularly relevant is the role of doxorubicin encapsulation, which does not compromise colloidal stability but seems to lead to further compaction of the particles. This effect is explained by the electrostatic interaction between the amino groups of doxorubicin and the carboxylic residues of the polyglutamate backbone within the NGs, with the drug molecules acting as nucleation centers for a denser and more homogeneous polymer network. This phenomenon opens the possibility of exploiting specific drug-polymer interactions or adding cationic artificial additives in the synthesis to achieve more controlled nucleation and to modulate carrier properties, thereby improving stability and controlled release from a therapeutic perspective. Microfluidic platforms have shown clear advantages over traditional batch methods in NGs synthesis, improving reproducibility, size uniformity, and encapsulation efficiency. For example, Majedi et al. produced chitosan-based NGs with narrow size distributions (100–200 nm) and high protein encapsulation efficiency [34], while Whiteley et al. optimized protein-loaded NGs with sizes around 84 nm and encapsulation efficiencies of up to 94.6% using microfluidics and “design-of-experiment” (DoE) approaches [35]. Compared to these studies, our PGA NGs show highly uniform sizes (75–105 nm, PDI 0.08–0.10) and reproducible drug loading (EE ~40%, DL ~0.90%), while offering a fully covalently crosslinked structure. The integration of microfluidics and SPAAC click chemistry enables mild reaction conditions compatible with sensitive payloads and rapid, continuous exploration of formulation parameters to fine-tune physicochemical properties. This approach overcomes the limitations of traditional batch synthesis, including high batch variability and difficulty in real-time process optimization, demonstrating the high reproducibility and tunability achievable with our platform [22,28]. Regarding the cited technical challenges in microfluidics, in our case: the use of a carefully controlled polymer mixture at a relatively low concentration (0.5% *w*/*w*, as reported in the Materials and Methods section) prevented clogging in all runs; all tubing and connections were made of PTFE, fully compatible with both water and acetone; we did not observe any effect attributable to non-specific adsorption on the device surfaces. Regarding the last point, it should be noted that the observed ~20% loss of polymers (most probably attributable to the purification steps rather than the microfluidic process itself) was lower than that in the batch synthesis (~35%) [31]. Finally, the modularity offered by microfluidics suggests the future integration of on-chip steps, such as automated purification and multi-step synthesis, which are crucial for efficient industrial-scale production.

Poly(α-L-glutamic acid) has previously been explored for the formulation of covalently crosslinked NGs capable of encapsulating doxorubicin, demonstrating promising stability and anticancer efficacy under physiological conditions [36]. However, such systems were typically produced via batch methods and lacked control over size tunability and scalability. In contrast, our approach integrates SPAAC click chemistry into a microfluidic framework, enabling the continuous production of covalently crosslinked PGA NGs with precise size control and reproducible drug loading, with EE% and DL% values of approximately 40% and 0.90%, respectively, consistent with the high polymer-to-drug ratio used. This allowed us to focus on particle size, uniformity, and reproducibility while maintaining stable drug incorporation.

Future perspectives thus focus on implementing automated optimization strategies and high-throughput screening for increasingly performant NG formulations, including the use of possible additives affecting nucleation and network compactness (e.g., cationic species). Expanding the platform to new crosslinking chemistries and bioactive payloads and experimenting with coprecipitation with high-molecular-weight compounds like proteins, oligomers, or ultrasmall nanoparticles represents a promising path to increase the versatility and applications of nanocarriers in nanomedicine. Moreover, targeted in vivo studies to assess the biodistribution, biocompatibility, and therapeutic efficacy of these nanoplatforms should be conducted. Overall, our work strengthens the established literature framework regarding the central role of process control in NGs design for drug delivery, emphasizing the value of an integrated microfluidics-click chemistry approach for the development of safer, more efficient, and scalable systems.

## 5. Conclusions

The development of advanced NGs platforms for biomedical applications requires not only innovative materials but also precise and scalable manufacturing strategies to address challenges in size distribution, encapsulation efficiency, and reproducibility, which are typical of traditional batch methods. In this study, we addressed these needs by establishing a microfluidic approach for the synthesis of poly(α-glutamic acid) (PGA) NGs using strain-promoted azide–alkyne cycloaddition (SPAAC) click chemistry as a highly efficient and biocompatible crosslinking method. By automatically modulating the microfluidic flow rates, we rapidly explored formulation parameters and fine-tuned the physicochemical properties of the NGs. Our results demonstrate that this approach enables the reproducible formation of PGA nanogels with highly uniform and tunable sizes ranging from approximately 75 to 105 nm, narrow size distributions (PDI 0.08–0.10), and controlled drug loading, with encapsulation efficiencies around 40%. The resulting nanogels were stable and robust, and capable of encapsulating sensitive therapeutic agents under mild reaction conditions.

These findings confirm that integrating microfluidic technology with SPAAC click chemistry overcomes common limitations of conventional batch synthesis, such as broad size distributions, variable encapsulation efficiencies, and limited scalability. The wide parameter space explored owing to the fast, automatic modulation of flow parameters within the microfluidic device allowed fine control over the physicochemical properties of NGs, most notably size, dispersion, and drug loading capacity, demonstrating the versatility and robustness of the platform. This tunability, demonstrated through our experimental results and supported by the literature on other hydrophilic polymer NGs [20,37,38], highlights the versatility and robustness of the system and its adaptability to diverse application requirements. While microfluidic synthesis and advanced crosslinking methods have been widely applied to NGs based on polymers like chitosan and hyaluronic acid, PGA-based NGs remain relatively underexplored.

Overall, our work lays a solid foundation for the next generation of NG technologies at the intersection of polymer chemistry, microfluidics, and biomedical engineering, offering a versatile and scalable platform for producing reproducible, customizable, and clinically translatable nanogels.

## Figures and Tables

**Table 1:** Summary of the experimental conditions used for NGs synthesis, including the individual flow rates of acetone and water, the calculated flow rate ratios (FRR), the corresponding total flow rates in mL/min (Flux TOT), and the reaction temperature (T). Alternate gray and white rows groups highlight conditions with the same Flux TOT.

Conditions	Acetone (mL/min)	H_2_0 (mL/min)	Ratio	Flux TOT	T (°C)
1	262.5	37.5	7	300	25
2	218.75	31.25	7	250	25
3	175	25	7	200	25
4	87.5	12.5	7	100	25
5	250	50	5	300	25
6	210	42	5	252	25
7	175	35	5	210	25
8	100	20	5	120	25
9	225	75	3	300	25
10	187.5	62.5	3	250	25
11	150	50	3	200	25
12	75	25	3	100	25
13	200	100	2	300	25
14	167	83.5	2	250.5	25
15	140	70	2	210	25
16	70	35	2	105	25
17	180	120	1.5	300	25
18	150	100	1.5	250	25
19	120	80	1.5	200	25
20	60	40	1.5	100	25
21	150	150	1	300	25
22	125	125	1	250	25
23	100	100	1	200	25
24	50	50	1	100	25
25	240	60	4	300	50
26	200	50	4	250	50
27	160	40	4	200	50
28	80	20	4	100	50

**Table 2:** Z-average of the diameter, polydispersity index (PDI), and, if applicable, encapsulation efficiency (EE%) for each nanogel (NG) formulation resulting from the conditions tested for NG synthesis as described in Table 1 and identified by the same number reported in the first column and by the same cell color, without or with encapsulation of doxorubicin (Dox). Data are shown as mean ± SD (*n* = 3). Standard deviations below 0.01 were replaced by a minimum value of 0.01, reflecting the estimated measurement accuracies. NGs Blank: empty nanogels; NGs Dox: Dox-loaded nanogels. NA (Not Achieved) indicates that particle formation did not occur under the tested conditions.

Conditions	NGs Blank		NGs Dox		
	Z-Average (nm) ± SD	PDI ± SD	Z-Average (nm) ± SD	PDI ± SD	EE (%) ± SD
1	145.5 ± 1.2	0.13 ± 0.03	193.1 ± 8.3	0.06 ± 0.06	54.8 ± 5.6
2	169.8 ± 6.6	0.14 ± 0.08	180.3 ± 4.1	0.07 ± 0.01	63.7 ± 14.2
3	170.7 ± 16.5	0.08 ± 0.04	159.4 ± 19.7	0.05 ± 0.01	58.4 ± 9.3
4	124.8 ± 24.3	0.09 ± 0.03	187.2 ± 9.5	0.08 ± 0.06	34.9 ± 10.4
5	81.5 ± 9.9	0.05 ± 0.02	70.3 ± 8.7	0.12 ± 0.02	28.3 ± 2.2
6	85.5 ± 4.7	0.05 ± 0.01	76.8 ± 6.1	0.07 ± 0.01	35.7 ± 8.1
7	91.4 ± 2.6	0.05 ± 0.02	80.2 ± 5.4	0.07 ± 0.01	36.3 ± 5.9
8	97.8 ± 4.0	0.05± 0.02	80.0 ± 9.3	0.07 ± 0.01	41.1 ± 7.7
9	92.2 ± 6.2	0.10 ± 0.04	62.5 ± 3.6	0.11 ± 0.06	37.2 ± 5.2
10	56.5 ± 5.5	0.09 ± 0.02	61.8 ± 3.1	0.09 ± 0.02	38.5± 6.5
11	59.3 ± 2.2	0.13 ± 0.02	56.1 ± 7.3	0.07 ± 0.03	40.0 ± 2
12	62.8 ± 5.0	0.09 ± 0.03	62.3 ± 9.3	0.10 ± 0.02	50.6 ± 14.3
13	55.0 ± 6.7	0.12 ± 0.04	47.3 ± 4.2	0.13 ± 0.01	20.8 ± 9.2
14	58.2 ± 4.0	0.10 ± 0.01	50.8 ± 3.7	0.14 ± 0.05	23.3 ± 4.2
15	58.0 ± 1.8	0.07 ± 0.02	52.9 ± 7.5	0.11 ± 0.01	29.8 ± 8.2
16	64.2 ± 0.7	0.11 ± 0.06	60.0 ± 9.9	0.11 ± 0.02	27.6 ± 7.3
17	65.7 ± 2.9	0.32 ± 0.08	58.1 ± 2.9	0.19 ± 0.01	12.0 ± 0.5
18	54.1 ± 9.0	0.26 ± 0.07	65.4 ± 1.8	0.21 ± 0.04	11.3 ± 2
19	55.5 ± 8.7	0.22 ± 0.05	69.0 ± 5.0	0.15 ± 0.04	12.8 ± 1.7
20	63.2 ± 10	0.19 ± 0.06	82.0 ± 5.1	0.22 ± 0.07	16.9 ± 1.9
21	NA				
22	NA				
23	NA				
24	NA				
25	143.6 ± 5.8	0.12 ± 0.03	77.0 ± 0.3	0.08 ± 0.05	31.2 ± 9.4
26	138.5 ± 4.8	0.11 ± 0.08	89.6 ± 6.3	0.09 ± 0.09	35.2 ± 7.9
27	143.0 ± 14.9	0.08 ± 0.04	111.4 ± 5.7	0.10 ± 0.04	40.6 ± 8.8
28	179.6 ± 7.5	0.10 ± 0.03	98.7 ± 4.8	0.06 ± 0.02	39.3 ± 8.6

## Data Availability

Data presented in this study is contained within the article. Further inquiries can be directed to the corresponding author.
